# PUNCH2: Explore the strategy for intrinsically disordered protein predictor

**DOI:** 10.1371/journal.pone.0319208

**Published:** 2025-03-26

**Authors:** Di Meng, Gianluca Pollastri

**Affiliations:** School of Computer Science, University College Dublin, Dublin, Ireland; National Institute of High Security Animal Diseases, INDIA

## Abstract

Intrinsically disordered proteins (IDPs) and their intrinsically disordered regions (IDRs) lack stable three-dimensional structures, posing significant challenges for computational prediction. This study introduces PUNCH2 and PUNCH2-light, advanced predictors designed to address these challenges through curated datasets, innovative feature extraction, and optimized neural architectures. By integrating experimental datasets from PDB (*PDB_missing*) and fully disordered sequences from DisProt (*DisProt_FD*), we enhanced model performance and robustness. Three embedding strategies—One-Hot, MSA-based, and PLM-based embeddings—were evaluated, with ProtTrans emerging as the most effective single embedding and combined embeddings achieving the best results. The predictors employ a 12-layer convolutional network (CNN_L12_narrow), offering a balance between accuracy and computational efficiency. PUNCH2 combines One-Hot, ProtTrans, and MSA-Transformer embeddings, while PUNCH2-light provides a faster alternative excluding MSA-based embeddings. PUNCH2 and its streamlined variant, PUNCH2-light, are competitive with other predictors on the CAID2 benchmark and rank as the top two predictors in the CAID3 competition. These tools provide efficient, accurate solutions to advance IDP research and understanding.

## Introduction

Intrinsically disordered proteins (IDPs) and intrinsically disordered regions (IDRs) are characterized by their lack of stable three-dimensional structures, which allows them to remain highly flexible [4]. This flexibility enables IDPs and IDRs to play critical roles in various biological processes, including signaling, regulation, molecular recognition, and diverse cellular functions [1,2]. However, their structural diversity presents significant challenges for both experimental observation and computational prediction [2].

The prediction of IDRs requires distinct strategies compared to structured regions due to their unique structural and functional properties. While structured region prediction has been extensively studied in protein computational research [5], IDR prediction remains a relatively new field with numerous challenges, both identified and emerging [19]. These challenges can be categorized into three key areas from a computational perspective: (a) the availability of high-quality databases, (b) effective feature extraction methods and network architectures, and (c) robust strategies for predictor evaluation [3].

**Lack of a comprehensive IDR database.** Several databases provide annotations for intrinsically disordered regions (IDRs). General annotation databases include DisProt [11], a community resource that curates high-quality IDR annotations based on literature, and MobiDB [13], which aggregates annotations from both experimental literature and computational predictors. Additionally, function-specific databases such as DIBS [15], MFIB [16], and PED [17] focus on IDRs related to specific biological functions or structural ensembles.

While MobiDB and DisProt are valuable resources for IDR analysis, their direct use in training IDR predictors is often limited. This limitation stems from inconsistencies in annotation quality across protein sequences. DisProt provides high-quality, literature-sourced annotations, but not all residues in a given sequence are annotated. On the other hand, MobiDB offers annotations for complete protein sequences, but the quality varies, combining experimental data with computational predictions. However, these databases are commonly used for evaluating predictors. For instance, the CAID challenges [19–22] utilized datasets where IDRs were defined based on DisProt annotations, while structured regions were derived from PDB data, explicitly excluding regions lacking experimental validation.

**IDR features extraction and network architecture design.** Protein sequences, composed of amino acids, must be converted into numerical representations or matrices for computational analysis. Theoretically, a protein sequence determines its structure, which in turn determines its function [11,13]. Protein sequence embedding techniques transform these sequences into fixed-dimensional matrices that encode key features. These features can include amino acid composition, sequence entropy, hydrophobicity, secondary structure predictions, disorder predictions, solvent accessibility, physicochemical properties, and evolutionary information. However, predictors may introduce biases, and experimental data are often unavailable. Consequently, biochemical features [41,42] and evolutionary information [43,44,46,49] are the most commonly used. Biochemical features are static, interpretable, and easily accessible, while evolutionary information, typically obtained through multiple sequence alignment (MSA) [24], is vital for understanding protein functions.

Despite its utility, MSA-based embeddings present significant challenges, particularly in IDR sequence embedding. MSA results generate a list of sequences similar to the query sequence, requiring additional analysis and complex model structures to capture hidden information and long-distance dependencies [43,46]. Common approaches involve combining MSA results with one-hot encoding or frequency-based representations, paired with machine learning methods like support vector machines (SVM) [36,44] or deep learning models like long short-term memory (LSTM) [43,45,46]. Hybrid architectures, such as convolutional neural networks (CNNs) [35] integrated with recurrent networks, such as cascaded Bidirectional Recurrent Neural Networks and Convolutional Neural Networks (CBRCNN) [47], have also been explored. However, MSA-based embeddings depend heavily on the availability of similar sequences, and their quality deteriorates when no comparable sequences exist—common in highly disordered proteins due to their lower conservation relative to structured regions [6]. Moreover, MSA embeddings are computationally intensive, time-consuming, and limited in capturing diverse, context-aware information, making them less suitable for IDR prediction tasks.

The emergence of large language models (LLMs), particularly Transformer architectures [48], has introduced an alternative in the form of Protein Language Models (PLMs) [27,28]. PLMs can capture complex patterns, such as sequential and contextual relationships, within extensive protein sequence datasets. They claim to encode biochemical, structural, and evolutionary features [28,29,31], learning directly from raw protein sequences or MSA results [30]. PLMs produce embeddings in a matrix format that is easier to interpret and generally faster and more informative than MSA embeddings. However, while PLMs offer significant potential, there is no conclusive evidence that they consistently outperform MSA-based methods in IDR prediction, nor has an optimal embedding approach for IDR prediction been established.

**Evaluation of IDR predictors.** Evaluating intrinsically disordered region (IDR) predictors presents several challenges. A significant issue is the potential bias in available datasets, which often emphasize well-studied proteins and organisms, limiting their ability to represent the full diversity of protein sequences and structures. Additionally, the dynamic nature of IDRs complicates evaluation, as these regions can exhibit context-dependent behavior—appearing ordered when interacting with partner proteins but disordered in isolation [7]. This variability makes consistent assessment of predictive accuracy difficult.

Another challenge lies in selecting appropriate performance metrics. Commonly used metrics such as accuracy, precision, recall, and F1 score are often insufficient for evaluating IDR predictors, as they may fail to account for the imbalanced nature of datasets, where disordered regions are typically less frequent than ordered regions [13,19]. These factors highlight the need for more tailored evaluation strategies to capture the complexities of IDR prediction effectively.

**Contributions of this work.** In this study, we aim to address these challenges by:

- Developing a systematic approach to IDR prediction, integrating diverse datasets, embedding methods, and network architectures.
- Evaluating the utility of PLM-based embeddings compared to traditional MSA-based methods in capturing IDR-specific features.
- Introducing robust predictors, PUNCH2 and PUNCH2-light, trained on curated datasets and evaluated on benchmark datasets from CAID challenges.

Through this work, we aim to provide both practical tools for IDR prediction and a comprehensive framework for building and evaluating IDR predictors, advancing the field toward more accurate and interpretable models.

## Proposed solution

Predicting intrinsically disordered regions (IDRs) requires tailored solutions due to their unique characteristics, such as their structural instability and context-dependent behavior. Existing predictors often adopt methods developed for structured protein regions, overlooking IDR-specific features. To address this, we propose a comprehensive framework that integrates curated datasets, advanced embedding techniques, neural architectures, and robust evaluation strategies.

To overcome the lack of a fully annotated IDR dataset, we curated a training set by combining experimentally derived sequences from *PDB_missing* with fully disordered sequences from DisProt (*DisProt_FD*). Benchmarking datasets were sourced from the Critical Assessment of Intrinsic Disorder (CAID) initiative, ensuring consistent and fair evaluation. The CAID2 dataset was selected as the primary benchmark due to its high-quality annotations derived from DisProt and PDB, while CAID1 and CAID3 datasets were incorporated to assess generalizability and historical context.

Embedding methods play a crucial role in capturing IDR-specific features. Two approaches were explored: (1) MSA-based embeddings, which leverage evolutionary information but require computationally intensive processing and are less effective for highly disordered sequences; and (2) PLM-based embeddings, which extract rich contextual features directly from protein sequences using Transformer-based architectures. PLM-based embeddings, such as ProtTrans and ESM-2, were evaluated for their ability to provide diverse and comprehensive feature representations.

For model architectures, neural networks were prioritized over traditional machine learning methods. Convolutional Neural Networks (CNNs) [35] were selected for their efficiency and ability to model local sequence patterns effectively. Various configurations were tested to optimize performance, including shallow and deep networks, as well as hybrid models combining CNNs with RNNs. The choice of architecture was guided by the need to balance computational efficiency with the ability to model complex sequence relationships.

Evaluation metrics were chosen to address the challenges of IDR prediction, particularly the imbalance between disordered and structured regions in datasets. Metrics such as AUC-ROC, APS, F1, MCC, and AUC-PR were utilized to provide a comprehensive assessment of predictor performance. These metrics were selected for their ability to capture performance across thresholds and provide insights into both precision and recall, ensuring a balanced evaluation of IDR predictors.

This proposed framework aims to address the limitations of existing methods by incorporating diverse datasets, leveraging state-of-the-art embeddings, and optimizing neural architectures. By aligning with CAID evaluation standards, this approach provides a robust foundation for developing IDR-specific predictors.

## Experiments

This study aims to develop robust predictors for IDRs by systematically exploring and integrating various datasets, embedding methods, and model architectures. The experiments were carefully designed to progress from foundational model selection to comprehensive evaluation on benchmarking datasets, ultimately leading to the development of two predictors, PUNCH2 and PUNCH2-light. This section outlines the dataset preparation, embedding methods, and network architectures employed in this study.

### Dataset preparation

**Fig 1:**
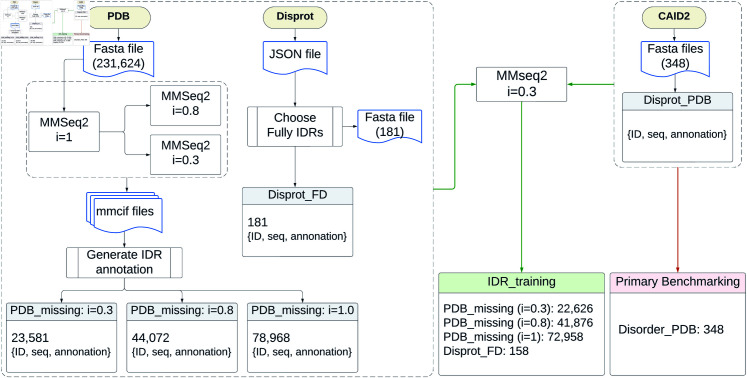
IDR data collection process. In the end, the IDR_Training dataset was searched against the Primary Benchmarking dataset (Disorder_PDB) by MMseqs2 with identity=0.3, and exclude the redundant sequences from the IDR_Training.

To train and evaluate our predictors, we curated multiple datasets categorized as training, validation, and benchmarking sets (Table 1 and Fig 1). The training datasets include the Primary Training Set (*PDB_missing: clstr30*), Extended Training Sets (*PDB_missing: clstr80* and *PDB_missing: clstr100*), and the Fully Disordered Supplementary Set (*DisProt_FD*). Benchmarking sets consist of the Primary Benchmarking Dataset (*Disorder_PDB*, CAID2), the Independent Benchmarking Dataset (*Disorder_PDB_3*, CAID3), and the Legacy Benchmarking Dataset (*Disorder_PDB_1*, CAID1).

**Table 1:** IDR dataset information.

Category	Dataset	Source	Usage	Num_Seq:Total	Num_Seq:Used	Description
Primary Training Set	PDB_missing: clstr30	PDB	Training & Validation	23,581	22,626	Generated with MMseq2 at 30% identity clustering. Missing residues from X-ray diffraction are annotated as disordered.
Fully Disordered Supplementary Set	DisProt_FD	DisProt	Training	181	158	Fully disordered proteins annotated in DisProt. Supplementary set to improve training representation of fully disordered regions.
Extended Training Sets	PDB_missing: clstr80	PDB	Training	44,072	41,876	Generated with MMseq2 at 80% identity clustering. Includes additional similar sequences to enrich training diversity.
	PDB_missing: clstr100	PDB	Training	78,968	72,958	Generated with MMseq2 at 100% identity clustering. Includes the largest number of sequences with only identical sequences removed.
Primary Benchmarking Dataset	Disorder_PDB	CAID2	Benchmarking	348	348	Evaluation dataset from CAID Round 2. Disordered residues are positives, observed residues are negatives, and unannotated regions are excluded.
Independent Benchmarking Dataset	Disorder_PDB_3	CAID3	Benchmarking	232	232	Recent benchmarking dataset from CAID Round 3. Used to evaluate model performance against the latest predictors.
Legacy Benchmarking Dataset	Disorder_PDB_1	CAID1	Benchmarking	652	652	Historical benchmarking dataset from CAID Round 1. Used to demonstrate consistency with older benchmarks.

Note: clstr30, clstr80, clstr100 represent sequence identity 0.3, 0.8, and 1.0 after MMseq2 clustering.

#### Benchmarking datasets.

The Primary Benchmarking Dataset (*Disorder_PDB*) was sourced from CAID2 [20]. This dataset consists of 348 sequences with conservative annotations: disordered residues are positive, observed residues from PDB are negative, and unannotated residues are excluded to avoid ambiguity. The Independent Benchmarking Dataset (*Disorder_PDB_3*) from CAID3 comprises 232 sequences, enabling comparisons with the latest predictors. Additionally, the Legacy Benchmarking Dataset (*Disorder_PDB_1*) from CAID1 includes 652 sequences, providing historical context. As other predictors’ results on CAID1 were obtained from the 2021 CAID1 benchmark, these results may not reflect updates to those predictors.

#### Training sets.

The training datasets were derived from PDB entries available as of July 26, 2023, using the query:

**Structure Determination Methodology = “experimental” AND (Experimental Method = “X-RAY DIFFRACTION” AND Polymer Entity Type = “Protein”)**.

This query yielded 231,624 entities. To ensure diverse and representative training data, 100% sequence identity clustering was performed, resulting in 78,968 unique sequences. These sequences were further clustered using MMseqs2 [23], producing datasets with varying sequence identity thresholds, *PDB_missing: clstr100* contains 78,968 sequences, *PDB_missing: clstr80* kept 44,072 representative sequences at 80% identity, and *PDB_missing: clstr30* kept 23,581 representative sequences at 30% identity.

Missing residues in X-ray diffraction data were labeled as disordered, with no length restrictions. These datasets allow us to explore the impact of dataset size and redundancy on training performance, balancing evolutionary diversity (*clstr30*) and data richness (*clstr100*).

**Fig 2:**
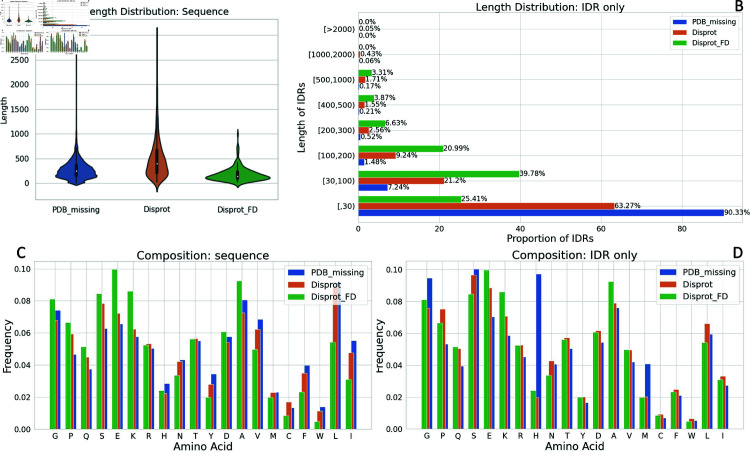
Dataset representation. IDRs from *PDB_missing: clstr30* serve as representative subsets for training, while *DisProt_FD* supplements with fully disordered sequences.

To address limitations in fully disordered sequences within the *PDB_missing* datasets, we curated the *DisProt_FD* dataset from DisProt [11], containing 181 sequences annotated as fully disordered. Comparative analysis (Fig 2) shows that *DisProt_FD* has a higher proportion of long IDRs compared to *PDB_missing*, complementing the training data.

To ensure test set independence, sequences in the training datasets with more than 30% identity to those in *Disorder_PDB* were excluded. This filtering yielded final training datasets combining *PDB_missing* clusters and *DisProt_FD*, while reserving *Disorder_PDB* exclusively for testing.

### Sequence embedding

This study employs eight embedding methods derived from three categories: One-Hot Encoding, MSA-Based Embedding, and PLM-Based Embedding. Detailed descriptions are provided in Table 2.

**Table 2:** Information for the 8 Embedding methods.

Name	Input	MAX-length	PLM	Embedding Dim	Description
One-Hot	Raw sequence	–	–	21	feature: 20(common AA) + 1(unusual)
MSA-prob	MSA result	–	–	22	feature: 20(common AA) + 1(unusual) + 1(Gap)
MSA-probAA	MSA result	–	–	22	feature: 20(common AA) + 1(unusual) + 1(Gap), set the AA probability in the sequence to 1
MSA-prob-numTemp	MSA result	–	–	23	feature: *MSA-prob* + 1 (number of templates from similarity searching)
MSA-probAA-numTemp	MSA result	–	–	23	feature: *MSA-probAA* + 1 (number of templates from similarity searching)
ESM-2	Raw sequence	1024	esm2-t33-650M-UR50D	1280	PLM trained on UR90, includes 650M params.
MSA Transformer	MSA result	1024	esm-msa1b-t12-100M-UR50S	768	PLM trained on UR50 + MSA, includes 100M params
ProtTrans	Raw sequence	–	ProtT5-XL-UniRef50	1024	PLM trained on UR50, includes 3B params

Note: The *ESM-2* and *MSA Transformer* models have a sequence length limitation of 1024 tokens, including the start and end tokens (i.e., 1022 sequence tokens plus a start token and an end token).

**One-hot encoding:** Each amino acid is represented as a sparse binary vector (1 × 21), with dimensions for 20 standard amino acids and one additional dimension for unusual residues. Despite its simplicity, it effectively retains sequence identity and is widely used in combination with other methods.

**MSA-based embedding:** Leveraging conserved regions across related proteins, MSA-based embeddings capture evolutionary signals. We used HHblits [26] to search the UniRef30 [18] database, generating probabilistic embeddings (*MSA-prob*) based on amino acid frequencies. Variants include *MSA-probAA* (exact residue probabilities set to 1) and *MSA-prob-numTemp* (template counts).

**PLM-based embedding:** Pre-trained models (*ProtTrans* [29], *ESM-2* [31], *MSA-Transformer* [30]) extract high-dimensional features from sequences. *ProtTrans* and *ESM-2* operate on raw sequences, while *MSA-Transformer* directly processes MSA results, embedding amino acids into 768-dimensional vectors.

### Network architecture

The network architecture is critical for effectively capturing the features encoded by different embeddings, which range from local sequence patterns to long-range dependencies.

**PrepBase** serves as a non-learning baseline, predicting IDRs based solely on amino acid frequencies derived from *PDB_missing: clstr30*. This dataset includes 3,972,049 residues, 10% of which are in IDRs. Using observed amino acid distributions (**S1 Table**), **PrepBase** maps each residue to its frequency without requiring training.

**StrucBase**, a baseline neural network, employs a single-layer CNN with a kernel size of 1, number of channels of 1, and *sigmoid* activation, processing residues independently without considering sequence context. This architecture efficiently handles variable-length inputs and provides a simple yet effective comparison point for more advanced models.

**Fig 3:**
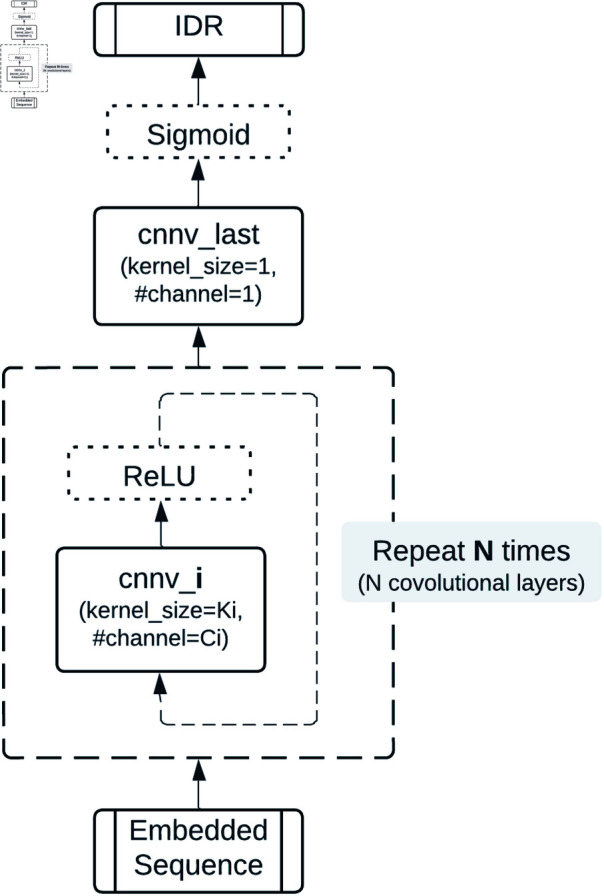
The structure of general CNN-based predictors. *N* is the total number of Convolutional layers, and *i* is the *i*th Convolutional layer.

Advanced architectures, including recurrent neural networks (RNNs) [32], LSTMs, and CNNs, were evaluated for their ability to capture sequential and local features. CNNs, in particular, demonstrated versatility in adapting to different embedding methods, as shown in Fig 3.

**Fig 4:**
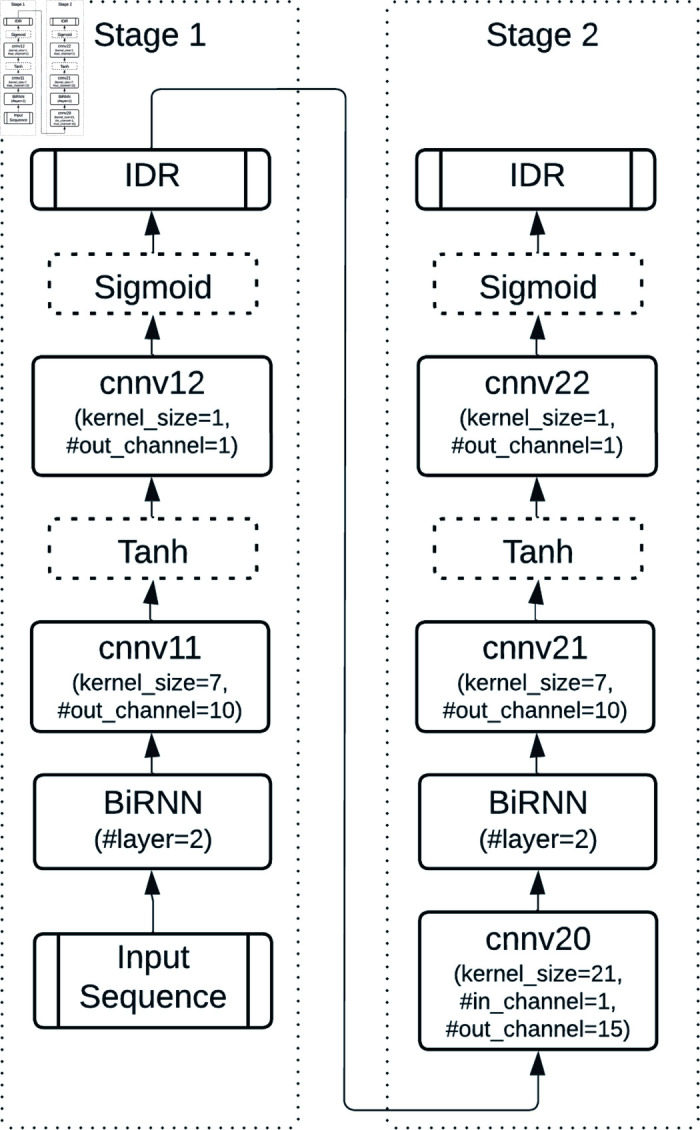
2-stage CBRCNN structure for IDR prediction. The 2 stages can be trained and evaluated separately.

To further improve performance, a modified CBRCNN architecture was implemented (Fig 4). CBRCNN combines CNN’s local feature extraction with RNN’s sequential modeling in a two-stage process: Stage 1 generates initial predictions, and Stage 2 refines these predictions using feedback from Stage 1. Both stages can be trained independently, offering flexibility and improved prediction accuracy.

These architectures were systematically evaluated to identify the most effective configurations for IDR prediction.

### Evaluation metrics

To assess the performance of our predictors, we utilized the following evaluation metrics:

*AUC-ROC*: Area Under the Receiver Operating Characteristic Curve, which evaluates the model’s ability to distinguish between classes across all thresholds.

*AUC-PR*: Area Under the Precision-Recall Curve, which focuses on the model’s performance in identifying the positive (disordered) class.

*APS*: Average Precision Score, a weighted mean of precision across recall levels, particularly useful for imbalanced datasets.

*F1 Score*: The harmonic mean of precision and recall, optimized for a specific threshold.

*MCC*: Matthews Correlation Coefficient, which provides a balanced measure of model quality, accounting for true positives, true negatives, false positives, and false negatives.

The formulas for these metrics are as follows:


$$\begin{array}{llll} \text{AUC-ROC Score}: & \;\text{AUC-ROC}=\int_{0}^{1}\text{TPR}({\text{FPR}}^{-1}(t))\;dt\; & & \;\\ \text{AUC-PR Score}: & \;\text{AUC-PR}=\int_{0}^{1}\text{Precision}({\text{Recall}}^{-1}(t))\;dt\; & & \;\\ \text{APS Score}: & \;\text{APS}=\frac{\overset{N}{\underset{k=1}{\sum }}({\text{Precision}}_{k}\cdot ({\text{Recall}}_{k}-{\text{Recall}}_{k-1}))}{N}\; & & \;\\ \text{F1 Score}: & \;\text{F1}=2\cdot \frac{\text{Precision}\cdot \text{Recall}}{\text{Precision}+\text{Recall}}\; & & \;\\ \text{MCC}: & \;\text{MCC}=\frac{\text{TP}\cdot \text{TN}-\text{FP}\cdot \text{FN}}{\sqrt{\left(\text{TP}+\text{FP})(\text{TP}+\text{FN})(\text{TN}+\text{FP})(\text{TN}+\text{FN}\right)}}\; & & \; \end{array}$$

### Confidence score

To enhance the interpretability of predictions, we introduced residue-level confidence scores. These scores reflect the reliability of predictions by integrating local sequence context through a smoothing operation. The confidence score $C_{i}$ for residue *i* is calculated as:


$$C_{i}=\{\begin{array}{cc} \frac{\frac{1}{w}\overset{i+\frac{w-1}{2}}{\underset{j=i-\frac{w-1}{2}}{\sum }}s_{j}-t}{1-t},\; & \text{if }\frac{1}{w}\overset{i+\frac{w-1}{2}}{\underset{j=i-\frac{w-1}{2}}{\sum }}s_{j}>t,\\ \frac{t-\frac{1}{w}\overset{i+\frac{w-1}{2}}{\underset{j=i-\frac{w-1}{2}}{\sum }}s_{j}}{t},\; & \text{if }\frac{1}{w}\overset{i+\frac{w-1}{2}}{\underset{j=i-\frac{w-1}{2}}{\sum }}s_{j}\leq t, \end{array}$$

where:

- $s_{j}$: Predicted disorder score for residue *j*,
- *t*: Threshold for disordered regions (*t* = 0.35),
- *w*: Sliding window size (odd, *w* = 3 by default).

Confidence values range from 0 to 1, with higher values indicating greater certainty in the prediction. Predictions near the threshold (*t*) exhibit lower confidence, while those near the extremes (*s* ≈ 0 or *s* ≈ 1) have higher confidence. These scores are independent of ground truth labels and serve as a measure of the model’s certainty, providing users with additional information for assessing predictions.

## Training process and results

The training process was structured into two phases to develop robust predictors for intrinsically disordered regions (IDRs). Phase 1 focused on identifying the best combinations of embedding methods and network architectures, referred to as “best singles”. Phase 2 involved incremental improvements and the creation of ensemble predictors. The outcomes of Phase 1 include several optimal model solutions (single predictors), while Phase 2 yielded the final predictors, PUNCH2 and PUNCH2-light. These predictors were trained on larger datasets and benchmarked on CAID2, CAID3, and CAID1 datasets for validation.

### Phase 1: Selecting the best model solutions

In Phase 1, the Primary Training Set (*PDB_missing: clstr30*) was divided into 70% for training and 30% for validation. The objective was to evaluate the performance of different embedding methods and network architectures using AUC-ROC as the primary evaluation metric.

#### Performance of embedding methods with baselines.

The baseline model **StrucBase** was trained using eight embedding methods: One-Hot, MSA-prob, MSA-probAA, MSA-prob-numTemp, MSA-probAA-numTemp, ProtTrans, ESM-2, and MSA Transformer. The results are summarized in Table 3.

**Table 3:** StrucBase: Performance of different embedding methods.

Embedding	Embedding Dim	#Parameters	#Train-Epoch	AUC-ROC
One-Hot	21	22	20	0.612
MSA-prob	22	23	400	0.835
MSA-probAA	22	23	300	0.833
MSA-prob-numTemp	23	24	300	0.846
MSA-probAA-numTemp	23	24	500	0.846
ESM-2	1280	1281	200	0.903
**MSA Transformer**	768	769	300	**0.921**
**ProtTrans**	1024	1025	900	**0.913**

Note: The performance of each embedding method combined with the baseline model StrucBase is reported using AUC-ROC scores.

PLM-based embeddings, particularly *ProtTrans* and *MSA Transformer*, consistently outperformed others. *MSA Transformer* excelled for sequences shorter than 1022 residues but required segmentation for longer sequences, resulting in suboptimal performance. *ProtTrans* outperformed *ESM-2*, while One-Hot encoding showed reasonable performance given its simplicity and computational efficiency. Among MSA-based methods, adding template-based features marginally improved results. Overall, PLM-based embeddings proved most effective for IDR prediction.

#### Network architectures.

**Fig 5:**
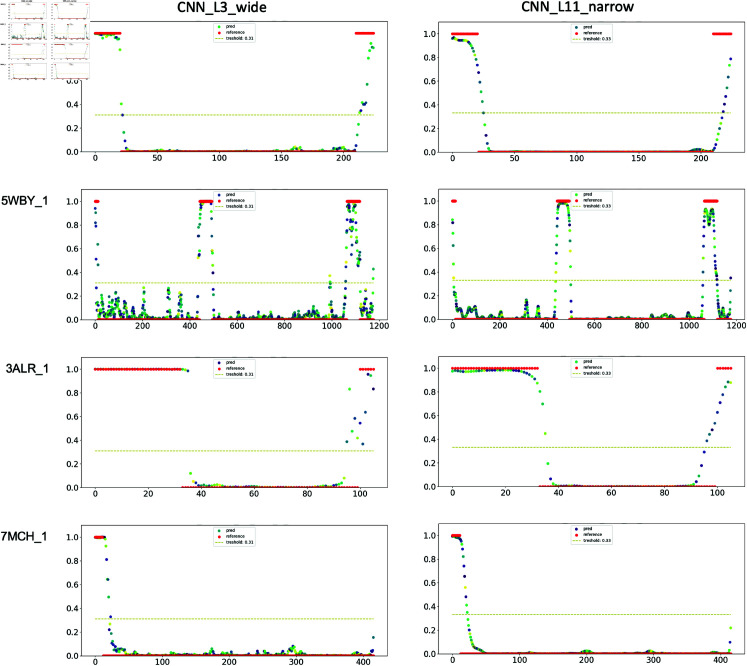
Comparison of CNN_L3_wide and CNN_L11_narrow using *ProtTrans* embedding. The architectures have approximately 157K and 180K parameters, respectively.

**CNN architectures: shallow vs. deep.** We evaluated CNN architectures by incrementally increasing complexity, including kernel size, number of channels, and depth. Two configurations emerged as optimal: **CNN_L11_narrow** (deeper and narrower, detailed structure in **S2 Table**) and **CNN_L3_wide** (shallower and wider, detailed structure in **S4 Table**). Both achieved comparable AUC-ROC scores but exhibited distinct characteristics. **CNN_L11_narrow** showed higher prediction confidence, as demonstrated in Fig 5.

**RNNs and LSTMs.** Recurrent models (RNNs and LSTMs) were tested to capture sequential dependencies, particularly for MSA-based embeddings. However, they failed to outperform CNNs. For instance, with *ProtTrans*, RNNs and LSTMs achieved an AUC-ROC of 0.92, compared to 0.93 for **CNN_L11_narrow**. Their longer training times further limited their scalability.

**Fig 6:**
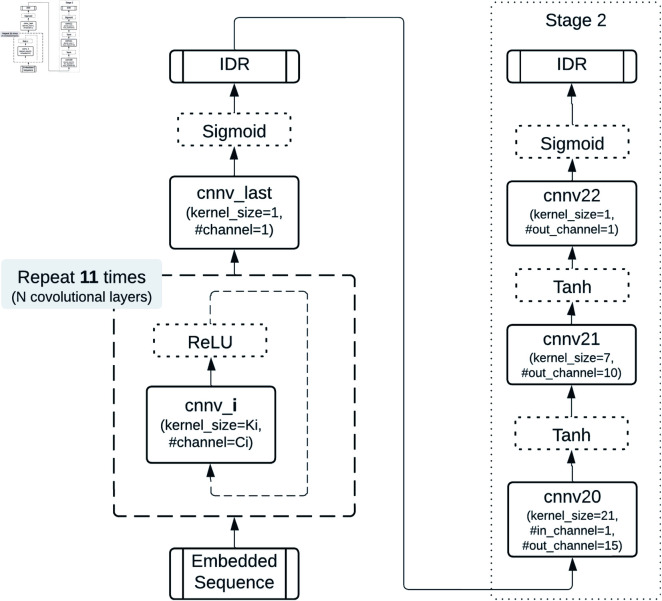
Two-stage CNN structure. Stage 1 corresponds to the best-performing architecture (CNN_L11_narrow), while Stage 2 adds a standalone CNN structure.

**CBRCNN and two-stage CNNs.** The CBRCNN model, which combines CNN and RNN components, achieved slightly better results than standalone RNNs, reaching an AUC-ROC of 0.925 when using *ProtTrans*. The two-stage CNN (Fig 6), which enhances **CNN_L11_narrow** by adding a second convolutional layer, did not lead to performance improvements. As shown in **S3 Fig**, the second convolutional stage did not improve the AUC; instead, the performance ultimately reached the same AUC as the first stage throughout the training process.

#### Best singles.

From the eight embedding-architecture combinations tested, three “best singles” were selected to capture diverse features for Phase 2 (Table 4):

- *One-Hot + CNN_L11_narrow*
- *ProtTrans + CNN_L11_narrow*
- *MSA-Transformer + CNN_L11_narrow*

**Table 4:** Optimal model solutions combining embeddings and network architectures.

Embedding	Network	#Layers	#Feature	#Param	AUC-ROC
**One-Hot**	CNN_L2	2	21	2,636	0.83
MSA-prob	CNN_L11_narrow	11	22	46,641	0.896
MSA-probAA	CNN3_L11_narrow	11	22	46,641	0.903
**ProtTrans**	CNN2_L3_wide	3	1,024	105,011	0.9306
	**CNN2_L11_narrow**	11	1,024	180,541	0.93
ESM-2	CNN2_L11_narrow	11	1,280	218,941	0.928
**MSA Transformer**	CNN2_L11_narrow	11	768	142,141	0.934

Note: *ProtTrans* has two optimal architectures: *CNN_L3_wide* and *CNN_L11_narrow*, but only *CNN_L11_narrow* was retained for Phase 2.

These selections represent three key embedding categories: One-Hot (simplicity), PLM-based (ProtTrans), and MSA-based (MSA-Transformer, which integrates PLM and MSA information). By selecting these models, we ensured a balanced representation of diverse embedding features in the next phase.

### Phase 2: Incremental improvements and ensemble formation

In Phase 2, the training process was expanded to include supplementary datasets (*DisProt_FD*, *PDB_missing: clstr80*, and *PDB_missing: clstr100*), and ensemble models were developed to combine the strengths of individual predictors. This phase focused on improving performance and robustness by leveraging a variety of models.

#### Step 1: Incorporating fully disordered sequences.

The training set was expanded by adding the Fully Disordered Supplementary Set (*DisProt_FD*) to the *PDB_missing: clstr30* dataset. The three selected best singles were retrained on this combined dataset, leading to improved performance on the Primary Benchmarking Dataset (*Disorder_PDB*, CAID2). This confirmed the added value of including fully disordered sequences, as seen in Table 5, where predictors 4, 6, and 7, trained on the expanded dataset, outperformed predictors 1, 2, and 3, respectively.

**Table 5:** Performance of predictors on *Disorder_PDB.*

Predictor	Dataset	Ensemble			AUC-ROC	APS	MaxF1	threshold
		One-Hot	MSA Transformer	ProtTrans				
1	PDB_missing: clstr30	1	0	0	0.878	0.796	0.712	0.08
2		0	1	0	0.941	0.886	0.807	0.19
3		0	0	1	0.932	0.878	0.80	0.25
4	PDB_missing: clstr30 +Disprot_FD	1	0	0	0.878	0.798	0.714	0.09
5		3	0	0	0.889	0.814	0.735	0.1
6		0	1	0	0.947	0.921	0.855	0.28
7		0	0	1	0.940	0.870	0.832	0.41
8		0	0	5	0.940	0.890	0.833	0.33
9		0	5	5	0.944	0.880	0.840	0.3
10		3	0	5	**0.943**	**0.900**	**0.834**	0.28
11		3	5	5	**0.947**	**0.910**	**0.844**	0.33
12	PDB_missing: clstr80 +2*Disprot_FD	3	5	5	0.948	0.912	0.846	0.36
13		3	0	5	0.945	0.905	0.841	0.36
14^1^	PDB_missing: clstr100 +3*Disprot_FD	3	5	5	**0.952**	**0.915**	**0.849**	0.378
15^2^		3	0	5	**0.95**	**0.911**	**0.845**	0.35

The numbers under Ensemble represent the number of predictors from each embedding method used in the ensemble. ^1^ PUNCH2. ^2^PUNCH2-light.

#### Step 2: Ensemble formation.

To enhance the robustness of the model, k-fold cross-validation was applied to each of the best single models:

- *One-hot + CNN_L11_narrow*: 3-fold cross-validation
- *ProtTrans + CNN_L11_narrow*: 5-fold cross-validation
- *MSA-Transformer + CNN_L11_narrow*: 5-fold cross-validation

This cross-validation process generated *k* single predictors per combination, capturing slight variations in training data while ensuring full coverage of the training set. Two ensemble predictors were formed based on this process:

- **Predictor 11** (Table 5) combines 3 predictors from *One-hot*, 5 from *ProtTrans*, and 5 from *MSA-Transformer*.
- **Predictor 10** (Table 5) combines 3 predictors from *One-hot* and 5 from *ProtTrans*, excluding MSA-based embeddings.

Both ensembles were evaluated on *Disorder_PDB* using AUC-ROC as the primary metric. **Predictor 11** achieved an AUC-ROC of 0.947, while **Predictor 10** achieved 0.943, demonstrating that the absence of MSA-based embeddings had minimal impact on performance.

#### Step 3: Scaling to larger datasets.

The analysis was scaled to larger datasets, *PDB_missing: clstr80 + 2x DisProt_FD* and *PDB_missing: clstr100 + 3x DisProt_FD*, and fine-tuned the **CNN_L11_narrow** architecture accordingly. The results were as follows:

1. Increasing the depth of **CNN_L11_narrow** from 11 to 12 layers (**CNN_L12_narrow**, **S3 Table**) yielded optimal performance across both larger datasets.
2. To maintain a similar emphasis on fully disordered sequences, *DisProt_FD* was repeated twice for *PDB_missing: clstr80* and three times for *PDB_missing: clstr100*.
3. The ensemble model, combining predictors trained on *PDB_missing: clstr100* augmented with three iterations of *DisProt_FD*, consistently demonstrated the best performance (Table 5).

The two best ensembles from Step 2 were retrained on these expanded datasets. Increasing dataset size further improved performance. Predictors trained on *PDB_missing: clstr100 + 3x DisProt_FD* yielded the best results. We named these predictors:

- PUNCH2: (One-Hot + ProtTrans + MSA-Transformer) @ CNN_L12_narrow on *PDB_missing: clstr100 + 3 DisProt_FD*.
- PUNCH2-light: (One-Hot + ProtTrans) @ CNN_L12_narrow on *PDB_missing: clstr100 + 3 DisProt_FD*.

#### Benchmarking results.

The final predictors, PUNCH2 and PUNCH2-light, were evaluated on the CAID2, CAID3, and CAID1 benchmarking datasets. Evaluation metrics included AUC-ROC, AUC-PR, MCC, F1, and APS, which provided a comprehensive assessment of prediction accuracy, precision, and robustness.

**Primary benchmarking (*Disorder_PDB*, CAID2).** PUNCH2 and PUNCH2-light demonstrated competitive performance on the *Disorder_PDB* dataset, achieving AUC-ROC scores of 0.951 and 0.950, respectively. These results rival SPOT-Disorder2, the top predictor in the CAID2 challenge, which achieved an APS of 0.928 (Table 6). Although SPOT-Disorder2 slightly outperformed in APS, PUNCH2 and PUNCH2-light delivered higher AUC-ROC and F1 scores, illustrating their robust performance across metrics.

**Table 6:** Performance comparison of PUNCH2, Top 10 CAID2 Predictors, and other well-known predictors on *Disorder_PDB.*

Index	Predictor	AUC-ROC	AUC-PR	APS	F1	Best_t	MCC
1	SPOT-Disorder2	0.949	0.929	0.928	0.860	0.361	0.795
2	AlphaFold-rsa	0.944	0.917	0.916	0.849	0.521	0.787
3	PredIDR-long	0.934	0.871	0.870	0.800	0.588	0.723
4	IDP-Fusion	0.933	0.878	0.878	0.822	0.488	0.756
5	SPOT-Disorder	0.931	0.889	0.889	0.823	0.374	0.758
6	SETH-0	0.930	0.894	0.893	0.830	0.413	0.771
7	AlphaFold-pLDDT	0.929	0.881	0.881	0.821	0.290	0.750
8	PredIDR-short	0.927	0.859	0.859	0.790	0.601	0.707
9	metapredict	0.923	0.878	0.877	0.819	0.484	0.756
10	DeepIDP-2L	0.922	0.858	0.858	0.794	0.361	0.710
11	IUPred3	0.885	0.825	0.825	0.746	0.444	0.646
12	AIUPred	0.903	0.855	0.855	0.776	0.629	0.695
13	ESpritz-D	0.899	0.810	0.810	0.757	0.248	0.659
14	ESpritz-N	0.859	0.766	0.766	0.696	0.317	0.574
15	ESpritz-X	0.882	0.794	0.792	0.713	0.053	0.599
16	MobiDB-lite	0.868	0.801	0.763	0.729	0.375	0.630
17	**PUNCH2**	**0.952**	**0.915**	**0.915**	**0.849**	**0.378**	**0.793**
18	**PUNCH2-light**	**0.950**	**0.912**	**0.911**	**0.845**	**0.350**	**0.787**

The numbers from 1 to 10 represent the performance rankings of various predictors in the CAID2 challenge. The last two entries, PUNCH2 and PUNCH2-light, are our newly developed predictors.

**Fig 7:**
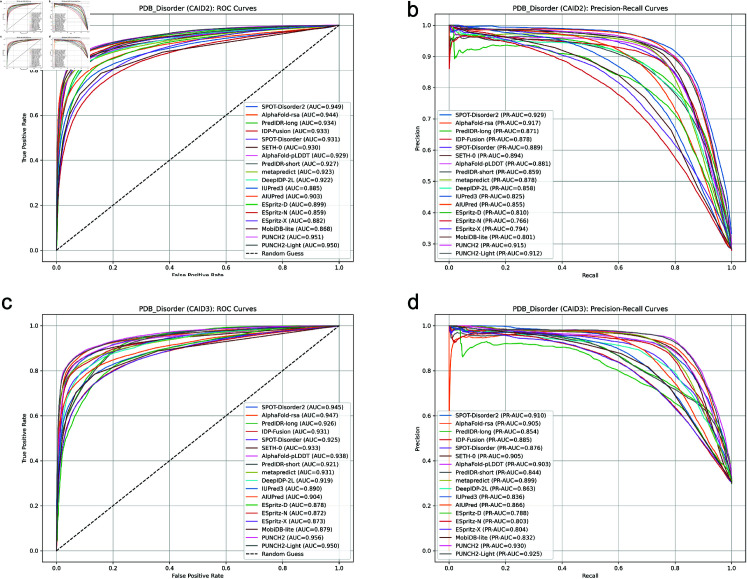
Performance on CAID2 and CAID3. ROC and PR curves for the performance of the predictors on *Disorder_PDB* (a&b, from CAID2) and *Disorder_PDB_3* (c&d, from CAID3).

**Independent benchmarking (*Disorder_PDB_3* from CAID3).** On the *Disorder_PDB_3* dataset, PUNCH2 achieved an AUC-ROC of 0.956, APS of 0.929, and F1 of 0.865, while PUNCH2-light achieved an AUC-ROC of 0.950, APS of 0.925, and F1 of 0.862. Both predictors outperformed SPOT-Disorder2 (AUC-ROC: 0.945, APS: 0.910, F1: 0.831) and AlphaFold-rsa (AUC-ROC: 0.947, APS: 0.905, F1: 0.851) (Table 7, Fig 7 parts c&d). These results highlight the robustness of PUNCH2 and PUNCH2-light in handling unseen datasets.

**Table 7:** Performance comparison of PUNCH2 and other well-known predictors on *Disorder_PDB_3* (CAID3).

Index	Predictor	AUC-ROC	AUC-PR	APS	F1	Best_t	MCC
1	SPOT-Disorder2	0.945	0.910	0.910	0.831	0.307	0.759
2	AlphaFold-rsa	0.947	0.905	0.905	0.851	0.558	0.790
3	PredIDR-long	0.926	0.854	0.853	0.762	0.528	0.655
4	IDP-Fusion	0.931	0.885	0.885	0.825	0.538	0.757
5	SPOT-Disorder	0.925	0.876	0.875	0.798	0.379	0.717
6	SETH-0	0.933	0.905	0.905	0.843	0.394	0.778
7	AlphaFold-pLDDT	0.938	0.903	0.902	0.841	0.296	0.773
8	PredIDR-short	0.921	0.844	0.844	0.757	0.533	0.647
9	metapredict	0.931	0.899	0.899	0.829	0.534	0.765
10	DeepIDP-2L	0.919	0.863	0.863	0.797	0.376	0.708
11	IUPred3	0.890	0.836	0.836	0.757	0.452	0.654
12	AIUPred	0.904	0.866	0.866	0.783	0.662	0.698
13	ESpritz-D	0.878	0.788	0.787	0.726	0.268	0.600
14	ESpritz-N	0.872	0.803	0.803	0.727	0.341	0.609
15	ESpritz-X	0.873	0.804	0.802	0.726	0.054	0.603
16	MobiDB-lite	0.879	0.832	0.793	0.757	0.400	0.664
17	**PUNCH2**	**0.956**	**0.930**	**0.929**	**0.865**	**0.384**	**0.806**
18	**PUNCH2-Light**	**0.950**	**0.925**	**0.925**	**0.862**	**0.352**	**0.802**

The numbers from 1 to 10 represent the performance rankings of various predictors in the CAID2 challenge. The last two entries, PUNCH2 and PUNCH2-light, are our newly developed predictors.

**Legacy benchmarking (*Disorder_PDB_1*, CAID1).** On the older *Disorder_PDB_1* dataset, PUNCH2 achieved an AUC-ROC of 0.939 and APS of 0.898, consistent with its performance on CAID2 and CAID3 datasets (Supplementary **S5 Table**). This demonstrates the stability of our predictors across diverse datasets.

#### Prediction result analysis.

To evaluate the predictors’ performance and applicability, we conducted statistical analysis and manual inspection of predictions on 71 fully annotated sequences from the *Disorder_PDB* dataset, including 29 fully disordered and 42 partially disordered sequences. The detailed statistical results are provided in the supplementary information (**S7 Table**, **S6 Table**, **S9 Table**, **S8 Table**, and **S2 Fig**). Key observations include:

- Overall Metrics: Across all 71 sequences, the average accuracy, true positive rate (TPR), and confidence score were 0.901, 0.73, and 0.795, respectively.
- Fully vs. Partially Disordered Sequences: For fully disordered sequences, the predictor achieved a TPR of 0.868, accuracy of 0.868, and confidence score of 0.68. For partially disordered sequences, accuracy (0.923) and confidence (0.874) were higher, but TPR (0.635) was lower, reflecting stronger overall predictions but a relative challenge in capturing true positives for partial disorder.
- Bias and Strengths: The predictor excelled in identifying terminal IDRs (e.g., sequence DP02757 in **S2 Fig**), while shorter internal IDRs were less accurately predicted, likely due to dataset bias favoring terminal regions.
- High Accuracy Consistency: Over 90% accuracy was achieved for 55 of 71 sequences, with 38 achieving TPR above 90%.

In summary, the predictors perform robustly, especially for fully disordered proteins and terminal IDRs, with high accuracy and confidence. However, predictions for internal IDRs remain a challenge, potentially linked to dataset imbalances. Manual inspection aligned with statistical analysis, reinforcing these conclusions.

## Conclusion

This study aimed to develop deep learning-based predictors for intrinsically disordered regions (IDRs) and to provide a detailed roadmap for constructing such predictors. By systematically exploring datasets, embedding methods, and network architectures, we developed two robust predictors: PUNCH2 and PUNCH2-light. These predictors were trained and evaluated on a combination of datasets, including experimentally derived sequences from PDB and fully disordered proteins from DisProt, with benchmarking performed on the CAID2, CAID3, and CAID1 datasets.

Our experiments highlighted the importance of incorporating fully disordered sequences (*DisProt_FD*) into the training process, significantly enhancing performance. These sequences contributed critical features absent in structured-region-focused datasets like *PDB_missing*, allowing our predictors to achieve robust predictions for both fully and partially disordered sequences. Datasets with 100% sequence identity (*PDB_missing: clstr100*) combined with larger models outperformed lower-identity datasets, suggesting that IDRs’ inherent sequence diversity enriches the training process while posing potential risks of dataset bias.

In the embedding evaluation, PLM-based methods, particularly *ProtTrans*, demonstrated superior performance compared to One-Hot encoding and MSA-based embeddings. Notably, *ProtTrans* offered stable and efficient predictions, slightly outperforming *ESM-2*. While *MSA-Transformer* excelled in specific cases, its dependency on multiple sequence alignment (MSA) and sequence length limitations made it less practical for high-throughput applications. Combining embeddings (One-Hot, ProtTrans, and optionally *MSA-Transformer*) proved optimal for capturing IDR-specific features.

From an architectural perspective, deeper convolutional neural networks (**CNN_L12_narrow**) consistently achieved high AUC scores and generated more confident predictions than shallower configurations, even with similar parameter counts. These architectures balanced capturing both local and long-range sequence features, providing the foundation for our final models.

PUNCH2 and PUNCH2-light embody the culmination of these efforts. Both predictors demonstrated strong performance, surpassing or matching top CAID2 predictors in key metrics such as AUC-ROC, APS, and F1. PUNCH2 combines One-Hot, ProtTrans, and *MSA-Transformer* embeddings, while PUNCH2-light omits *MSA-Transformer* for computational efficiency with minimal accuracy loss. These predictors, trained solely on sequence-based features, are straightforward and broadly applicable for IDR prediction tasks. Their simplicity ensures accessibility for researchers seeking to predict IDRs or replicate and build upon our work. Both tools, PUNCH2 and PUNCH2-light, are publicly available on GitHub (https://github.com/deemeng/punch2 and https://github.com/deemeng/punch2_light, respectively). Additionally, the datasets used in this project can be accessed on Hugging Face (https://huggingface.co/datasets/deeeeeeeeee/PUNCH2_data).

However, PUNCH2 and PUNCH2-light have limitations. The predictors were primarily trained on datasets derived from X-ray crystallography, where missing residues are annotated as IDRs. This introduces a bias toward shorter terminal IDRs and fully disordered proteins while underrepresenting internal and longer IDRs. Our analysis confirmed that the predictors perform well on termini and fully disordered sequences but are less accurate for internal IDRs. Additionally, as ensemble models, they provide robust and stable performance but reduce interpretability compared to single predictors.

To address these limitations and further enhance the predictors, future work could include:

- Incorporating NMR-derived mobile regions from PDB.
- Expanding training data with fully annotated DisProt datasets.
- Adding functional IDR-specific databases, such as disordered linkers and binding regions.
- Extending predictions to include functional annotations of IDRs.

In conclusion, PUNCH2 and PUNCH2-light offer practical and effective tools for IDR prediction, combining state-of-the-art performance with computational efficiency. This work also provides a detailed blueprint for developing IDR predictors, offering insights into dataset design, embedding selection, and network architecture optimization. While limitations remain, these predictors establish a strong foundation for future advancements in IDR prediction and functional analysis.

## Supporting information

S1 Table PrepBase: probability model. PrepBase specifies the probability distribution for each amino acid. The probabilities are calculated based on the residue composition from the PDB_missing: clstr30 dataset. This distribution serves as a foundational reference for the model, helping to encode the inherent likelihood of each amino acid’s occurrence.
(XLSX)

### Model hyperparameters

The model structures for three main CNN architectures are detailed below, each with specific configurations and hyperparameters.

S2 Table Model structure of CNN_L11_narrow. CNN_L11_narrow consists of 11 layers with a narrow configuration. The model employs a learning rate of 0.0001. The narrower architecture is designed to focus on extracting detailed features through multiple convolutional layers, allowing the model to capture subtle patterns in the data. #features stands for the number of input features. For instance, if the embedding method used is One-Hot, the #feature=21.
(XLSX)

S3 Table Model structure of CNN_L12_narrow. This table outlines the structure of **CNN_L12_narrow**, a variant with an additional convolutional layer compared to **CNN_L11_narrow**. This model also uses a learning rate of 0.0001, aiming to further refine feature extraction for a larger dataset through increased depth, potentially capturing more complex hierarchical features. #features stands for the number of input features. For instance, if the embedding method used is One-Hot, the #feature=21.
(XLSX)

S4 Table Model structure of CNN_L3_wide. **CNN_L3_wide** consists of 3 layers but with a wider configuration, meaning each layer has more filters. The wider structure allows for a broader capture of features at each level, facilitating the recognition of diverse patterns within the input data. This model also operates with a learning rate of 0.0001. #features stands for the number of input features. For instance, if the embedding method used is One-Hot, the #feature=21. These different configurations highlight the exploration of depth versus breadth in CNN architectures, providing insights into the trade-offs between layer depth and layer width in capturing features from protein sequences.
(XLSX)

### Benchmarking

S5 Table Performance comparison of PUNCH2 and other well-known predictors on Disorder_PDB_1 (CAID1). The performance of PUNCH2 on the CAID1 dataset (*Disorder_PDB_1*) was compared against other well-known predictors. It summarizes these results, highlighting the robustness of PUNCH2 in this historical benchmarking context.
(XLSX)

S1 Fig ROC and PR curves for the performance of the predictors on *Disorder_PDB_1* (a&b, from CAID1). (TIF)

#### Prediction analysis on CAID2

The performance of PUNCH2 and PUNCH2-light was further analyzed on the CAID2 dataset (*Disorder_PDB*). Specifically, we focused on 71 fully annotated sequences, comprising 29 fully disordered proteins and 42 partially disordered proteins.

**S6 Table** presents the performance of PUNCH2 on the 42 partially disordered protein sequences, sorted by accuracy, while **S7 Table** provides a similar analysis for the 29 fully disordered sequences. Correspondingly, **S8 Table** and **S9 Table** summarize the prediction performance of PUNCH2-light on the same datasets. These tables highlight the consistent performance of both predictors across different categories of sequences.

Additionally, **S2 Fig** illustrates specific examples of PUNCH2 predictions. For fully disordered proteins such as DP03738 and DP02911, PUNCH2 achieved high prediction accuracy, while it struggled with sequences like DP03489. Similarly, for partially disordered proteins, PUNCH2 performed well on terminus-located IDRs, as seen in sequences DP03610 and DP03622, but showed lower accuracy for internal IDRs, such as those in DP03647. This observation underscores the challenges in predicting internal IDRs and highlights the strengths of PUNCH2 in handling terminus-located disordered regions.

S6 Table Prediction performance of PUNCH2 for 42 partially disordered protein sequences, sorted by accuracy. (XLSX)

S7 Table Prediction performance of PUNCH2 for 29 fully disordered protein sequences, sorted by accuracy. (XLSX)

S8 Table Prediction performance of PUNCH2-light for 42 partially disordered protein sequences, sorted by accuracy. (XLSX)

S9 Table Prediction performance of PUNCH2-light for 29 fully disordered protein sequences, sorted by accuracy. (XLSX)

S2 Fig PUNCH2 prediction examples. DP03738, DP02911, and DP03489 are fully disordered proteins. PUNCH2 performs very well on DP03738 and DP02911 but poorly on DP03489. DP03647, DP03610, and DP03622 are partially disordered proteins. PUNCH2 performs very well on DP03610 and DP03622 but struggles with DP03647. The results also demonstrate that PUNCH2 is particularly effective at predicting terminus-located IDRs, whereas internal IDRs are more challenging.
(TIF)

S3 Fig Training progress of CBRCNN. *AUC_S1* denotes the AUC scores for Stage 1 and *AUC_S2* for Stage 2.
(TIF)

## References

[1] Alberts B, Johnson A, Lewis J. Analyzing Protein Structure and Function. In: Chapter 3. New York: Garland Science; 2002. Available from: https://www.ncbi.nlm.nih.gov/books/NBK26820/

[2] van der Lee R, Buljan M, Lang B, Weatheritt RJ, Daughdrill GW, Dunker AK, et al. Classification of intrinsically disordered regions and proteins. Chem Rev. 2014;114(13):6589–631. doi: 10.1021/cr400525m 24773235 PMC4095912

[3] Walsh I, Fishman D, Garcia-Gasulla D, Titma T, Pollastri G, ELIXIR Machine Learning Focus Group, et al. DOME: recommendations for supervised machine learning validation in biology. Nat Methods. 2021;18(10):1122–7. doi: 10.1038/s41592-021-01205-4 34316068

[4] Dunker AK, Babu MM, Barbar E, Blackledge M, Bondos SE, Dosztányi Z, et al. What’s in a name? Why these proteins are intrinsically disordered: why these proteins are intrinsically disordered. Intrinsically Disord Proteins 2013;1(1):e24157. doi: 10.4161/idp.24157 28516007 PMC5424803

[5] Kryshtafovych A, Schwede T, Topf M, Fidelis K, Moult J. Critical assessment of methods of protein structure prediction (CASP)-Round XV. Proteins. 2023;91(12):1539–49. doi: 10.1002/prot.26617 37920879 PMC10843301

[6] Brown C, Johnson A, Daughdrill G. Evolutionary rate heterogeneity in proteins with long disordered regions. J Mol Evol. 2011;73(3–4):229–36. doi: 10.1007/s00239-011-9476-z 12165847

[7] Dyson HJ, Wright PE. Intrinsically unstructured proteins and their functions. Nat Rev Mol Cell Biol. 2005;6(3):197–208. doi: 10.1038/nrm1589 15738986

[8] Torrisi M, Pollastri G. Brewery: deep learning and deeper profiles for the prediction of 1D protein structure annotations. Bioinformatics. 2020;36(12):3897–8. doi: 10.1093/bioinformatics/btaa204 32207516

[9] Armstrong DR, Berrisford JM, Conroy MJ, Gutmanas A, Anyango S, Choudhary P, et al. PDBe: improved findability of macromolecular structure data in the PDB. Nucleic Acids Res. 2020;48(D1):D335–43. doi: 10.1093/nar/gkz990 31691821 PMC7145656

[10] Berman HM, Westbrook J, Feng Z, Gilliland G, Bhat TN, Weissig H, et al. The Protein Data Bank. Nucleic Acids Res. 2000;28(1):235–42. doi: 10.1093/nar/28.1.235 10592235 PMC102472

[11] Aspromonte MC, Nugnes MV, Quaglia F, Bouharoua A, DisProt Consortium, Tosatto SCE, et al. DisProt in 2024: improving function annotation of intrinsically disordered proteins. Nucleic Acids Res. 2024;52(D1):D434–41. doi: 10.1093/nar/gkad928 37904585 PMC10767923

[12] Sickmeier M, Hamilton JA, LeGall T, Vacic V, Cortese MS, Tantos A, et al. DisProt: the Database of Disordered Proteins. Nucleic Acids Res. 2007;35(Database issue):D786-93. doi: 10.1093/nar/gkl893 17145717 PMC1751543

[13] Piovesan D, Necci M, Escobedo N, Monzon AM, Hatos A, Mičetić I, et al. MobiDB: intrinsically disordered proteins in 2021. Nucleic Acids Res. 2021;49(D1):D361–7. doi: 10.1093/nar/gkaa1058 33237329 PMC7779018

[14] Kumar M, Michael S, Alvarado-Valverde J, Mészáros B, Sámano-Sánchez H, Zeke A, et al. The Eukaryotic Linear Motif resource: 2022 release. Nucleic Acids Res. 2022;50(D1):D497–508. doi: 10.1093/nar/gkab975 34718738 PMC8728146

[15] Schad E, Fichó E, Pancsa R, Simon I, Dosztányi Z, Mészáros B. DIBS: a repository of disordered binding sites mediating interactions with ordered proteins. Bioinformatics. 2018;34(3):535–7. doi: 10.1093/bioinformatics/btx640 29385418 PMC5860366

[16] Fichó E, Reményi I, Simon I, Mészáros B. MFIB: a repository of protein complexes with mutual folding induced by binding. Bioinformatics. 2017;33(22):3682–4. doi: 10.1093/bioinformatics/btx486 29036655 PMC5870711

[17] Ghafouri H, Lazar T, Del Conte A, Tenorio Ku LG, PED Consortium, Tompa P, et al. PED in 2024: improving the community deposition of structural ensembles for intrinsically disordered proteins. Nucleic Acids Res. 2024;52(D1):D536–44. doi: 10.1093/nar/gkad947 37904608 PMC10767937

[18] UniProt Consortium. UniProt: the Universal Protein Knowledgebase in 2023. Nucleic Acids Res. 2023;51(D1):D523–31. doi: 10.1093/nar/gkac1052 36408920 PMC9825514

[19] Necci M, Piovesan D, CAID Predictors, DisProt Curators, Tosatto SCE. Critical assessment of protein intrinsic disorder prediction. Nat Methods. 2021;18(5):472–81. doi: 10.1038/s41592-021-01117-3 33875885 PMC8105172

[20] Conte AD, Mehdiabadi M, Bouhraoua A, Miguel Monzon A, Tosatto SCE, Piovesan D. Critical assessment of protein intrinsic disorder prediction (CAID) - results of round 2. Proteins. 2023;91(12):1925–34. doi: 10.1002/prot.26582 37621223

[21] Del Conte A, Bouhraoua A, Mehdiabadi M, Clementel D, Monzon AM, CAID predictors, et al. CAID prediction portal: a comprehensive service for predicting intrinsic disorder and binding regions in proteins. Nucleic Acids Res. 2023;51(W1):W62–9. doi: 10.1093/nar/gkad430 37246642 PMC10320102

[22] CAID Challenge Results. Critical Assessment of Intrinsic Disorder (CAID). [cited 2024 Dec 19]. Available from: https://caid.idpcentral.org/challenge/results

[23] Steinegger M, Söding J. Clustering huge protein sequence sets in linear time. Nat Commun 2018;9(1):2542. doi: 10.1038/s41467-018-04964-5 29959318 PMC6026198

[24] Needleman SB, Wunsch CD. A general method applicable to the search for similarities in the amino acid sequence of two proteins. J Mol Biol. 1970;48(3):443–53. doi: 10.1016/0022-2836(70)90057-4 5420325

[25] Altschul SF, Madden TL, Schäffer AA, Zhang J, Zhang Z, Miller W, et al. Gapped BLAST and PSI-BLAST: a new generation of protein database search programs. Nucleic Acids Res. 1997;25(17):3389–402. doi: 10.1093/nar/25.17.3389 9254694 PMC146917

[26] Steinegger M, Meier M, Mirdita M, Vöhringer H, Haunsberger SJ, Söding J. HH-suite3 for fast remote homology detection and deep protein annotation. 2019. doi: 10.1101/560029 PMC6744700 31521110

[27] Valentini G, Malchiodi D, Gliozzo J, Mesiti M, Soto-Gomez M, Cabri A, et al. The promises of large language models for protein design and modeling. Front Bioinform. 2023;3:1304099. doi: 10.3389/fbinf.2023.1304099 38076030 PMC10701588

[28] Schmirler R, Heinzinger M, Rost B. Fine-tuning protein language models boosts predictions across diverse tasks. Nat Commun 2024;15(1):7407. doi: 10.1038/s41467-024-51844-2 39198457 PMC11358375

[29] Elnaggar A, Heinzinger M, Dallago C, Rehawi G, Wang Y, Jones L, et al. ProtTrans: toward understanding the language of life through self-supervised learning. IEEE Trans Pattern Anal Mach Intell. 2022;44(10):7112–27. doi: 10.1109/TPAMI.2021.3095381 34232869

[30] Rao R, Liu J, Verkuil R, Meier J, Canny J, Abbeel P, et al. MSA transformer. bioRxiv. 2021. doi: 10.1101/2021.02.12.430858

[31] Lin Z, Akin H, Rao R, Hie B, Zhu Z, Lu W, et al. Language models of protein sequences at the scale of evolution enable accurate structure prediction. bioRxiv. 2022. Available from: https://www.biorxiv.org/content/early/2022/04/10/2022.04.10.487578

[32] Elman JL. Finding structure in time. Cognit Sci. 1990;14(2):179–211. doi: 10.1207/s15516709cog1402_1

[33] Hochreiter S, Schmidhuber J. Long short-term memory. Neural Comput. 1997;9(8):1735–80. doi: 10.1162/neco.1997.9.8.1735 9377276

[34] Baldi P, Brunak S, Frasconi P, Soda G, Pollastri G. Exploiting the past and the future in protein secondary structure prediction. Bioinformatics. 1999;15(11):937–46. doi: 10.1093/bioinformatics/15.11.937 10743560

[35] Luo Y, Uzuner Ö, Szolovits P. Bridging semantics and syntax with graph algorithms-state-of-the-art of extracting biomedical relations. Brief Bioinform 2017;18(4):722. doi: 10.1093/bib/bbx048 28472242 PMC6080366

[36] Cortes C, Vapnik V. Support-vector networks. Mach Learn. 1995;20(3):273–97. doi: 10.1007/bf00994018

[37] Erdős G, Dosztányi Z. AIUPred: combining energy estimation with deep learning for the enhanced prediction of protein disorder. Nucleic Acids Res. 2024;52(W1):W176–81. doi: 10.1093/nar/gkae385 38747347 PMC11223784

[38] Han K-S, Song S-R, Pak M-H, Kim C-S, Ri C-P, Del Conte A, et al. PredIDR: Accurate prediction of protein intrinsic disorder regions using deep convolutional neural network. Int J Biol Macromol. 2025;284(Pt 1):137665. doi: 10.1016/j.ijbiomac.2024.137665 39571839

[39] Emenecker RJ, Griffith D, Holehouse AS. Metapredict: a fast, accurate, and easy-to-use predictor of consensus disorder and structure. Biophys J. 2021;120(20):4312–9. doi: 10.1016/j.bpj.2021.08.039 34480923 PMC8553642

[40] Lotthammer JM, Hernández-García J, Griffith D, Weijers D, Holehouse AS, Emenecker RJ. Metapredict enables accurate disorder prediction across the Tree of Life. 2024. doi: 10.1101/2024.11.05.622168

[41] Dosztányi Z, Csizmók V, Tompa P, Simon I. The pairwise energy content estimated from amino acid composition discriminates between folded and intrinsically unstructured proteins. J Mol Biol. 2005;347(4):827–39. doi: 10.1016/j.jmb.2005.01.071 15769473

[42] Erdős G, Pajkos M, Dosztányi Z. IUPred3: prediction of protein disorder enhanced with unambiguous experimental annotation and visualization of evolutionary conservation. Nucleic Acids Res. 2021;49(W1):W297–303. doi: 10.1093/nar/gkab408 34048569 PMC8262696

[43] Hanson J, Paliwal KK, Litfin T, Zhou Y. SPOT-Disorder2: improved protein intrinsic disorder prediction by ensembled deep learning. Genom Proteom Bioinform. 2019;17(6):645–56. doi: 10.1016/j.gpb.2019.01.004 32173600 PMC7212484

[44] Vullo A, Bortolami O, Pollastri G, Tosatto SCE. Spritz: a server for the prediction of intrinsically disordered regions in protein sequences using kernel machines. Nucleic Acids Res. 2006;34(Web Server issue):W164-8. doi: 10.1093/nar/gkl166 16844983 PMC1538873

[45] Walsh I, Martin AJM, Di Domenico T, Vullo A, Pollastri G, Tosatto SCE. CSpritz: accurate prediction of protein disorder segments with annotation for homology, secondary structure and linear motifs. Nucleic Acids Res. 2011;39(Web Server issue):W190-6. doi: 10.1093/nar/gkr411 21646342 PMC3125791

[46] Walsh I, Martin AJM, Di Domenico T, Tosatto SCE. ESpritz: accurate and fast prediction of protein disorder. Bioinformatics. 2012;28(4):503–9. doi: 10.1093/bioinformatics/btr682 22190692

[47] Torrisi M, Kaleel M, Pollastri G. Deeper profiles and cascaded recurrent and convolutional neural networks for state-of-the-art protein secondary structure prediction. Sci Rep 2019;9(1):12374. doi: 10.1038/s41598-019-48786-x 31451723 PMC6710256

[48] Vaswani A, Shazeer N, Parmar N, Uszkoreit J, Jones L, Gomez AN, Kaiser L, Polosukhin I. Attention is all you need. arXiv preprint 2023. https://arxiv.org/abs/1706.03762

[49] Liu Y, Wang X, Liu B. A comprehensive review and comparison of existing computational methods for intrinsically disordered protein and region prediction. Brief Bioinform. 2019;20(1):330–46. doi: 10.1093/bib/bbx126 30657889

