# Supplementary figures and images for: PUNCH2: Explore the strategy for intrinsically disordered protein predictor
Source: PLoS One. 2025 Mar 26;20(3):e0319208. doi: 10.1371/journal.pone.0319208 (PMC11940444; doi:10.1371/journal.pone.0319208)

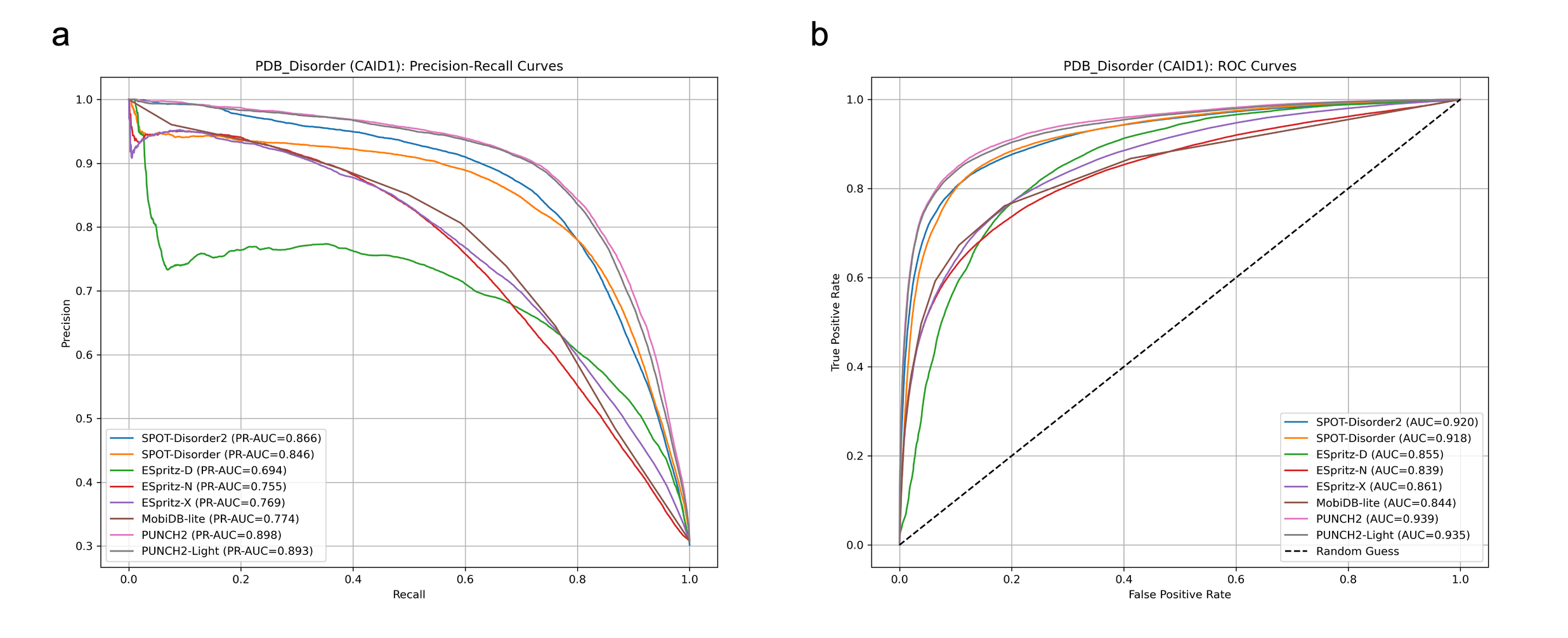

Supplement: S1 Fig — (TIF) [file pone.0319208.s006.tif]

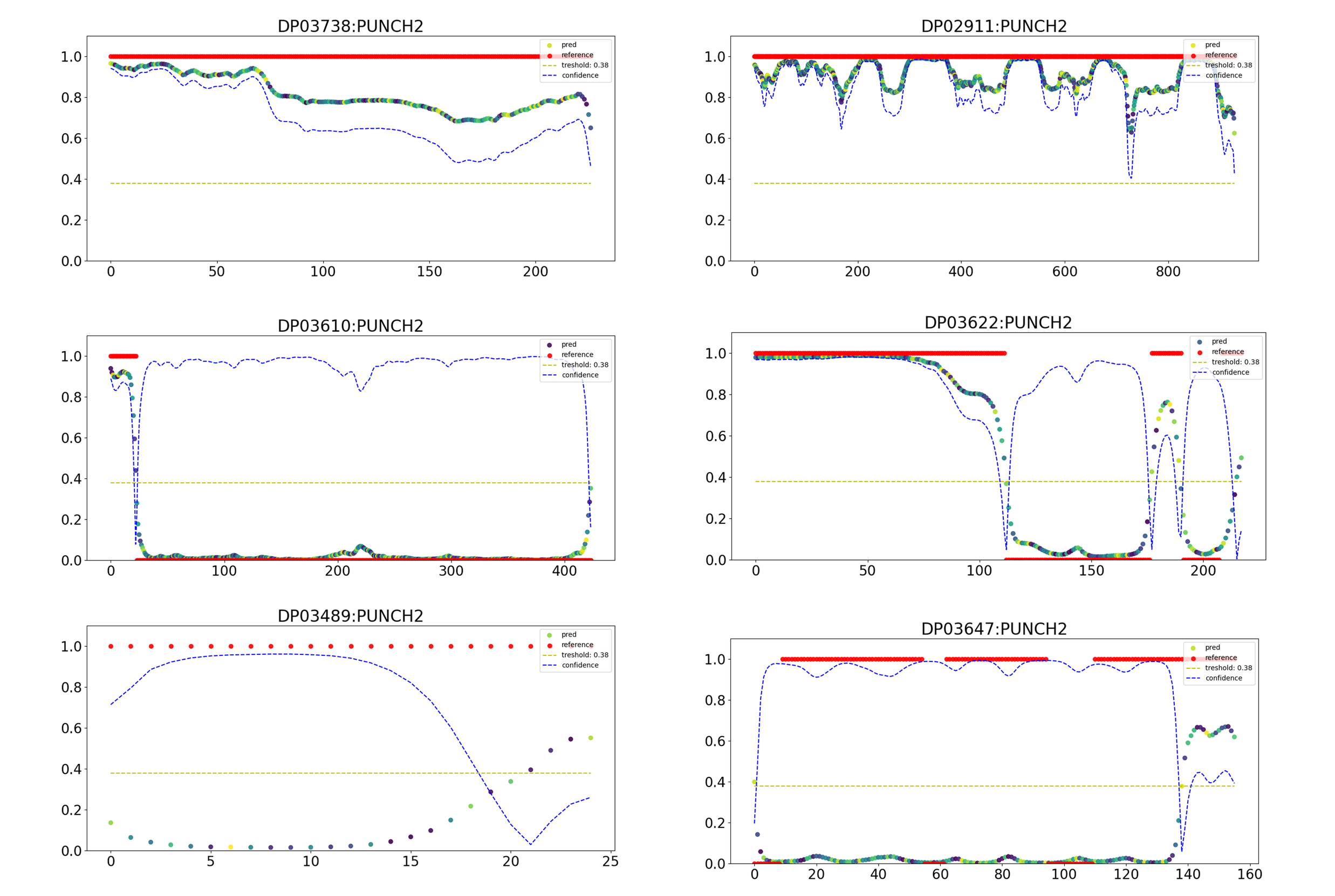

Supplement: S2 Fig — DP03738, DP02911, and DP03489 are fully disordered proteins. PUNCH2 performs very well on DP03738 and DP02911 but poorly on DP03489. DP03647, DP03610, and DP03622 are partially disordered proteins. PUNCH2 performs very well on DP03610 and DP03622 but struggles with DP03647. The results also demonstrate that PUNCH2 is particularly effective at predicting terminus-located IDRs, whereas internal IDRs are more challenging. (TIF) [file pone.0319208.s011.tif]

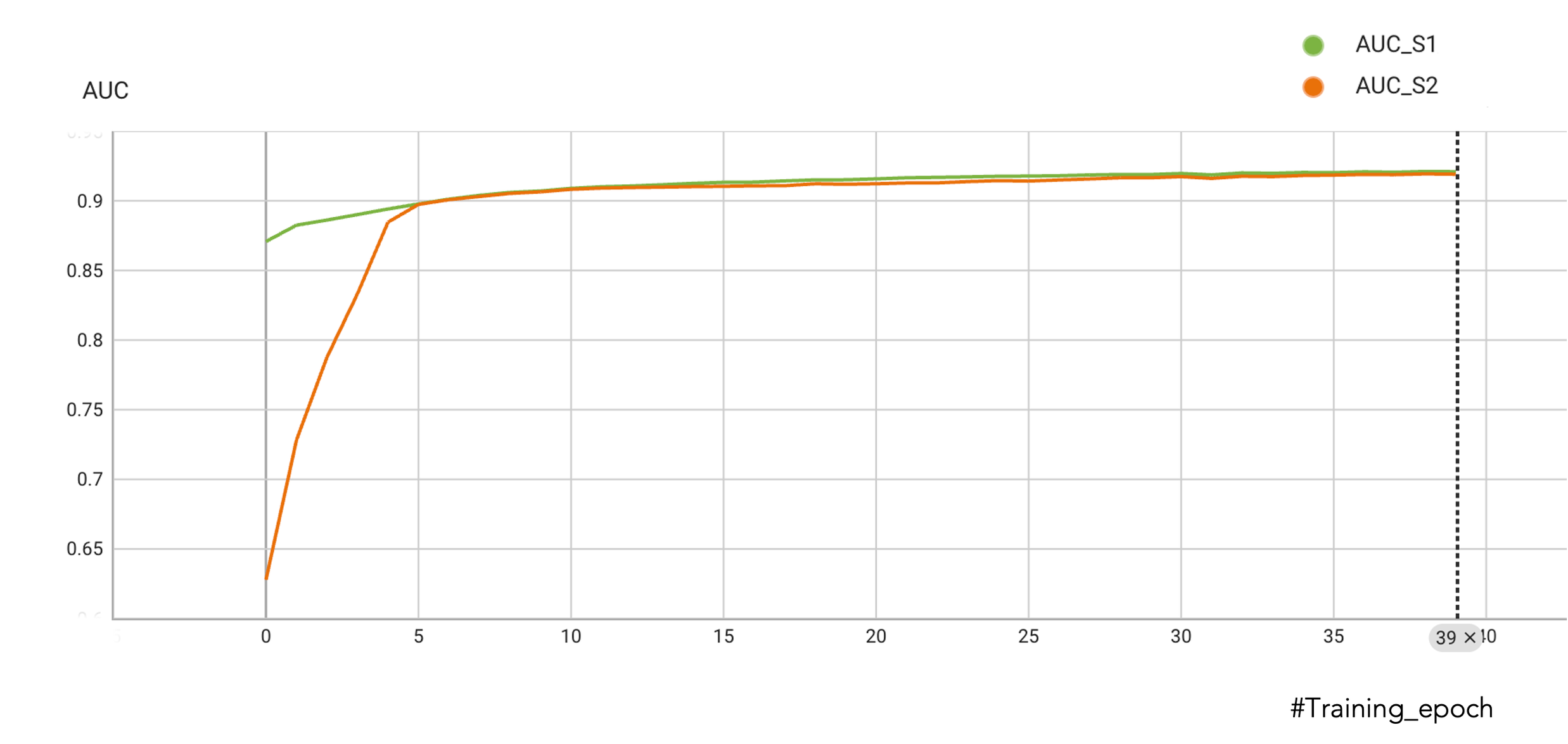

Supplement: S3 Fig — AUC_S1 denotes the AUC scores for Stage 1 and AUC_S2 for Stage 2. (TIF) [file pone.0319208.s012.tif]
